# Preparation and Mechanism Analysis of Stainless Steel AOD Slag Mixture Base Materials

**DOI:** 10.3390/ma17050970

**Published:** 2024-02-20

**Authors:** Liuyun Huang, Guogao Wei, Zhuxin Lan, Yuliang Chen, Tun Li

**Affiliations:** School of Civil Engineering and Architecture, Guangxi University of Science and Technology, Liuzhou 545006, China; 100000635@gxust.edu.cn (L.H.); 13098778576@163.com (Z.L.); ylchen@gxust.edu.cn (Y.C.); 100000644@gxust.edu.cn (T.L.)

**Keywords:** semirigid base material, stainless steel AOD slag, strength, modulus, microscopic mechanism analysis

## Abstract

To promote resourceful utilization of argon oxygen decarburization (AOD) slag, this research developed a new three-ash stabilized recycled aggregate with AOD slag, cement, fly ash (FA), and recycled aggregate (RA) as raw materials. The AOD slag was adopted as an equal mass replacement for fly ash. The application of this aggregate in a road base layer was investigated in terms of its mechanical properties and mechanistic analysis. First, based on a cement: FA ratio of 1:4, 20 sets of mixed proportion schemes were designed for four kinds of cement dosage and AOD slag replacement rates (*R*/%). Through compaction tests and the 7-day unconfined compressive strength test, it was found that a 3% cement dosage met the engineering requirements. Then, the unconfined compressive strength test, indirect tensile strength test, compressive rebound modulus test, and expansion rate test were carried out at different age thresholds. The results showed that the mixture’s strength, modulus, and expansion rate increased initially and then stabilized with age, while the strength and modulus initially increased and then decreased with increasing *R*. Secondly, based on X-ray diffraction (XRD) and scanning electron microscopy (SEM) used to analyze the mechanism, it was found that the strength, modulus, and expansion rate of the new material can be promoted by blending AOD slag, due to its ability to fully stimulate the hydration reaction and pozzolanic reaction of the binder. Finally, based on the strength and modulus results, *R* = 3% was identified as the optimal ratio, which provides a reference point for the effective application of AOD slag and RA in road base materials.

## 1. Introduction

In recent years, with the continuous development of economic construction and infrastructure, annual solid waste emissions, such as stainless steel slag and waste concrete blocks, have increased. China’s stainless steel and crude steel production have ranked among the top in the world. According to incomplete statistics, national stainless steel and crude steel production in the first half of 2022 equated to approximately 16.354 million tons [1]. This continuously increasing level of production has caused an accumulation of stainless steel slag produced in the smelting process of crude stainless steel, and the resulting waste of resources and environmental pollution problems have become increasingly prominent [2]. It has been pointed out that approximately one ton of waste residue is produced for every three tons of stainless steel [3]. At the same time, large numbers of waste concrete blocks are produced during the construction or demolition of various buildings. Therefore, the need for efficient treatment and recycling of stainless steel slag and waste concrete has become an urgent problem to be solved [4,5].

The composition of stainless steel slag mainly includes electric arc furnace (EAF) slag and argon oxygen decarburization (AOD) slag [6]. Among them, AOD slag is produced in the refining process, forming two states of solid and powder during cooling. It is conducive to collection and processing and has reasonable prospects for recycling in concrete engineering and road engineering. In an earlier study, Rosales et al. [7] analyzed the composition of AOD slag and found that its composition was similar to ordinary Portland cement and that it had certain cementitious activity [8,9,10]. Adegoloye et al. [11,12] used AOD slag instead of natural aggregate in concrete and found that it can slightly improve the mechanical properties of concrete. However, in using AOD slag as an aggregate, there may be a risk of heavy metal leaching under long-term erosion caused by rainwater, which poses a potential safety hazard to the environment. For this reason, Wu et al. [13] turned to using AOD slag to prepare cement and cementitious materials, and its compressive strength met the requirements for P.O. 32.5 ordinary Portland cement. Then, Gupta et al. [14,15] used AOD slag to partially replace the cement content in concrete pavement. Although the compressive strength decreased slightly, it also met the design requirements for rigid pavement, indicating its potential as an inorganic binder for road base. Chen et al. [16] used stainless steel slag instead of fly ash to prepare a three-ash gravel mixture. They found that its anti-erosion performance and dynamic compressive resilience modulus were significantly enhanced. Zhu et al. [17] tested the strength, water stability, and frost resistance of lime, fly ash, and stainless steel slag-stabilized gravel mixtures, which were found to meet the requirements for highway bases in China.

At the same time, to accelerate the sustainable development of resources, there is also a current research hotspot surrounding the crushing and screening of the waste concrete blocks produced by building demolition into recycled aggregates, which are then applied in road engineering [18,19,20,21]. Teijon et al. [22] and Garach et al. [23] first confirmed the feasibility of recycled aggregate as a road base. Then, Chiranjeevi et al. [24] and Tefa et al. [25] tested the road performance of a cement-stabilized recycled aggregate mixture and found that it had good compressive strength and could be used for low-grade roads. Due to the low mechanical properties of cement-stabilized recycled aggregate, some scholars have used waste brick slag aggregate as a supplementary material to evaluate the mixture’s road performance and fatigue characteristics, which were found to have good mechanical properties and durability [26,27,28]. Subsequently, Li et al. [29] studied the road performance of cement fly ash-stabilized steel slag and recycled aggregate mixtures and found that their strength meets the requirements for high-grade road bases in China. Baldo et al. [30] made 100% waste material (metallurgical slag) into foamed asphalt mixture and studied its indirect tensile strength, stiffness modulus, and fatigue resistance. The results showed that the foamed asphalt stable mixture has good performance as a road base. Rondinella et al. [31] used construction and demolition waste and recycled asphalt pavement as raw materials to prepare asphalt mixtures for reuse in road pavements. The results showed that both the volume of voids and the indirect tensile strength met the critical values for road material specifications.

In summary, the experimental study of AOD slag in concrete proves that it has certain cementitious properties and can be used as cementitious material for road base. Although the performance of recycled aggregate (RA) is poor, mixtures that meet road base specifications can be prepared through a reasonable mix design. The application of AOD slag, RA, and other environmentally friendly renewable resources to road construction, while ensuring that road performance requirements are met, conforms to the concept of green development and informs the direction of road construction development in the future. In terms of the current research on the application of AOD slag and RA as materials in road base materials, there have been few reports. For this reason, this study takes a cement fly ash (FA) composite cementitious system as the basis [32,33,34]. This study adopts AOD slag as an equal mass replacement of FA cement to constitute a FA–AOD slag composite cementitious system, which is then supplemented with recycled concrete aggregate as the stabilizing material, to form a new type of three-ash stabilized RA. This research studied the mixture’s physical and mechanical properties at different ages, and the mechanism of action was explored from a micro perspective. Finally, combined with the application requirements for road base materials, the optimal ratio was determined. The research results provide reference ideas and a basis for the comprehensive treatment and utilization of stainless steel AOD slag and RA as road base materials.

## 2. The Main Raw Materials Used in the Experiment

### 2.1. Cement

The cement used was Yufeng P.O. 42.5 ordinary Portland cement produced by Guangxi Yufeng Cement Co., Ltd. The performance of the cement was tested according to the method in standard JTG 3420-2020 [35]. The mass ratio of cement to ISO standard sand is 1:3, and the w/c ratio is 0.5. The indicators meet the relevant requirements for standard JTG/T F20-2015 [36]. The main performance indicators are shown in Table 1.

### 2.2. Fly Ash

The fly ash used was grade II fly ash produced by Henan Platinum Run Casting Material Co., Ltd. (Zhengzhou, China). The performance of the fly ash was tested according to the method in standard JTG E51-2009 [37]. The technical indicators met the relevant requirements for standard JTG/T F20-2015 [36]. The main performance indicators are shown in Table 2.

### 2.3. The Stainless Steel AOD Slag

**Figure 1:**
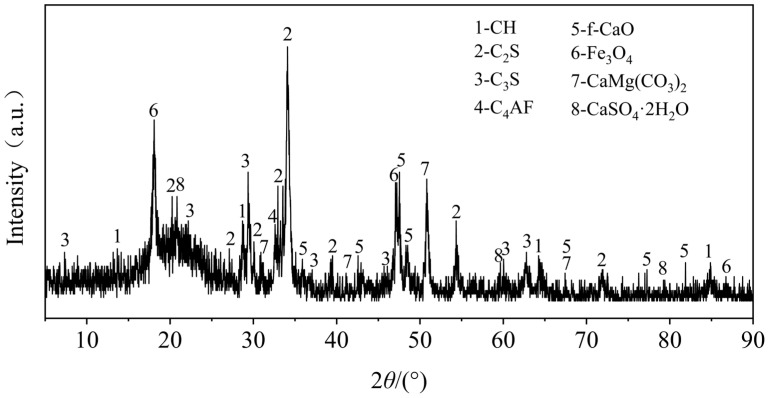
XRD pattern of stainless steel slag.

**Figure 2:**
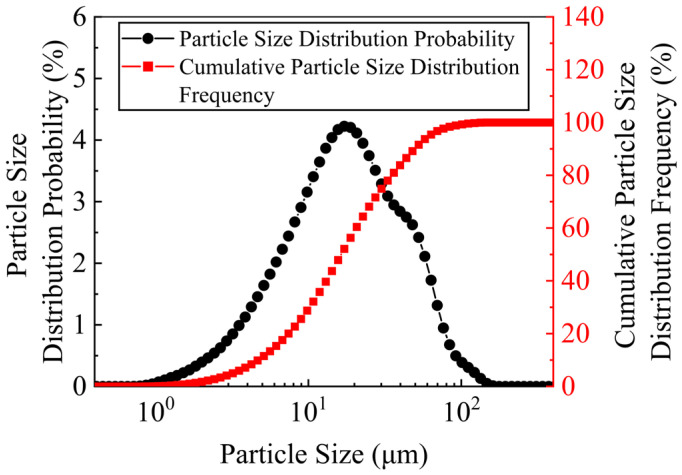
Grain size frequency distribution curve and cumulative grain size distribution curve of stainless steel slag.

Stainless steel slag powder produced by Guangxi Liuzhou Iron and Steel (Group) Company (Liuzhou, China) was used in the test. It is a stainless steel AOD slag obtained by the harmless treatment of solid waste produced in the later stages of stainless steel refining. Its appearance is grayish-white with powdered fine particles and a loose structure. The mineral composition and chemical composition of the stainless steel AOD slag were determined using X-ray diffraction (XRD) and X-ray fluorescence (XRF). The main mineral components are shown in Figure 1. The main mineral components in the AOD slag were found to be tricalcium silicate (C_3_S), 21%; dicalcium silicate (C_2_S), 34%; iron ore (Fe_3_O_4_), 10%; and iron aluminum acid four calcium (C_4_AF), 7%. The peaks of the C_3_S and C_2_S were more prominent than the other minerals. The main chemical composition is shown in Table 3, and is similar to the mineral and chemical composition of cement [38], which is the main reason for the activity of AOD slag. Figure 2 shows the particle size distribution of stainless steel AOD slag analyzed using a laser particle size analyzer. The characteristic particle size is 24.77 μm, D_10_ = 5.15 μm, D_50_ = 18.02 μm, and D_90_ = 54.35 μm, indicating that the particle group of stainless steel AOD slag is fine. A particle size below 40 μm greatly influences mineral activity [39]; the cumulative content of stainless steel AOD slag particle sizes below 43.67 μm is 86.56%, indicating that its potential activity is good. 

### 2.4. The Recycled Aggregate

The recycled aggregate is a waste concrete produced by a construction site in Liuzhou. It was broken by a jaw crusher and screened using a 30 mm square hole sieve. The particle size of the coarse aggregate is a continuous gradation of 4.75~31.5 mm, and the particle size of the fine aggregate is a continuous gradation of 0.075~4.75 mm. The performance test for the recycled aggregate was carried out according to the method in standard JTG E42-2005 [40]. All of the technical indicators met the relevant requirements for standard JTG/T F20-2015 [36]. The main performance characteristics are shown in Table 4 and Table 5.

## 3. Mixture Design and Test Method

### 3.1. Gradation Design

To avoid the influence of aggregate gradation differences on the strength of the mixture, only one aggregate gradation was used in the test. According to the aggregate gradation of the relevant requirements in standard JC/T 2281-2014 [41], the cement fly ash-stabilized recycled aggregate was selected for the median value of the particle composition of the base, as shown in Table 6. 

### 3.2. Proportioning Mix Design

According to the requirements for standard JTG/T F20-2015 [36], cement content should be selected in the range of 3% to 5%. Since the physical and mechanical properties of the RA are much worse than those of natural gravel, cement dosages of 3%, 4%, 5%, and 6% were selected. However, the ratio between binders is cement:FA = 1:3~1:5, and the ratio between the binder and stabilized material is cement and FA:stabilized material = 15:85~20:80. Therefore, this test selected a cement:FA ratio of 1:4. To study the effective application of stainless steel AOD slag in road base, by adopting AOD slag as an equal mass replacement of FA, a replacement rate of *R*/%, with cement, FA, and AOD slag as the binder, and RA as the stabilizing material, the strength, modulus, and expansion rate of the mixture were tested. The mix design is shown in Table 7.

### 3.3. Experimental Methods

According to the compaction test method recommended by T 0804-1994 in standard JTG E51-2009 [37], the maximum dry density (MDD) and optimum water content (OWC) of the AOD slag mixture were obtained. On this basis, a cylindrical specimen with a size of 150 mm × 150 mm was made, and the compaction degree was 98%. Each group had five parallel specimens. The UCS of standard conservation for 7 days under different cement dosages was tested to determine the cement dosage that met the engineering requirements. Furthermore, the UCS, ITS, and CRM of the AOD slag mixture at different ages were tested to determine the optimal ratio. Under the engineering cement dosage, according to standard G/BT 24175-2009 [42], the high-temperature water bath expansion rate test was carried out for 10 days, and the optimal ratio test piece was observed for 90 days. 

## 4. Test Results and Analysis

### 4.1. Compaction Test

**Figure 3:**
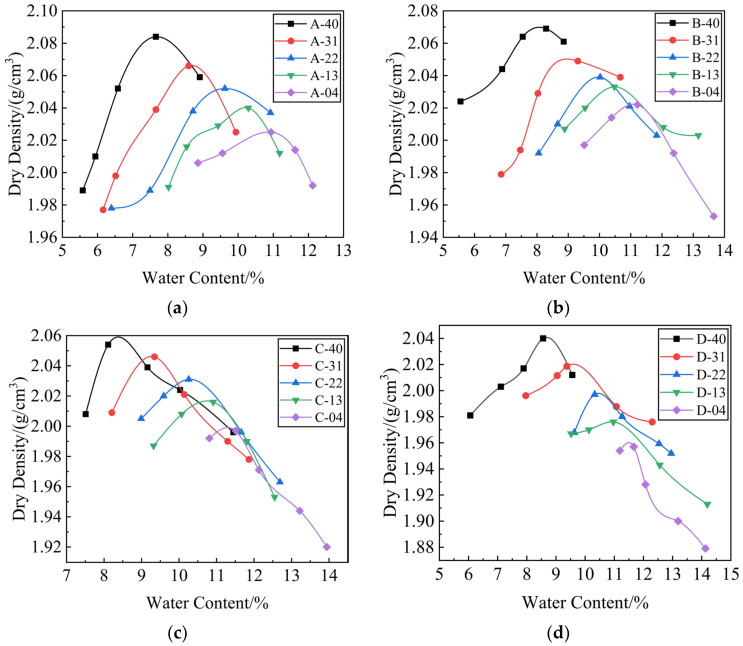
Compaction test curve of stainless steel AOD slag mixture. (**a**) The relationship between water content and dry density for the mixture with a 3% cement dosage; (**b**) the relationship between water content and dry density for the mixture with a 4% cement dosage; (**c**) the relationship between water content and dry density for the mixture with a 5% cement dosage; (**d**) the relationship between water content and dry density for the mixture with a 6% cement dosage.

Figure 3 shows the relationship curve between the water content and dry density of the stainless steel AOD slag mixture specimens at different cement dosages. The dry density of the 20 groups of stainless steel AOD slag mixtures increased first, and then decreased with the increase in water content. At the same cement dosage, the OWC of the mixture increased with the increase in the replacement rate *R* of stainless steel AOD slag, and the MDD decreased with the increase in the replacement rate *R* of stainless steel AOD slag. The OWC of the mixture was maintained between 7% and 12%, and the MDD was maintained between 1.96 and 2.09. The specific data are shown in Table 8.

### 4.2. Unconfined Compressive Strength (UCS)

**Figure 4:**
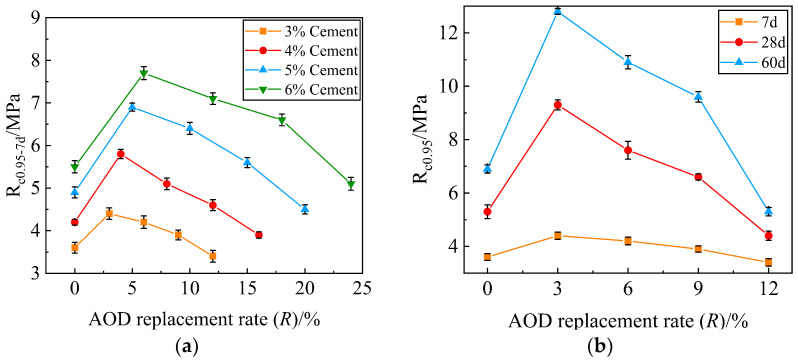
Unconfined compressive strength. (**a**) The 7-day unconfined compressive strength of stainless steel AOD slag mixture at different cement contents; (**b**) unconfined compressive strength of stainless steel AOD slag mixture at different ages.

Figure 4a shows the variation curve of the 7-day UCS representative value R_c0.95-7d_ with AOD slag replacement rate *R* under different cement dosages. The 7d UCS values of the four groups of cement doses all meet the requirements for specification JTG/T F20-2015 [36]. To save costs, a 3% cement dosage was determined to meet the engineering application requirements. Figure 4b shows the curve of UCS representative value R_c0.95_ with the AOD slag replacement rate *R* at different ages at the 3% cement dosage. From that figure, the following can be seen:

1. At different cement dosages, the distribution characteristics of the 7d UCS change curve of the mixture specimen are similar; the UCS value increases with the increase in cement content. The minimum 7d UCS values of the four cement dosage mixture specimens are all greater than 3 MPa, which meets the requirements for standard JTG/T F20-2015 [36] specifying that the cement–FA-stabilized base material not be less than 2.5 MPa. When *R* = 3%, *R* = 4%, *R* = 5%, and *R* = 6%, the UCS values of the four groups of cement dosage specimens reached their peak. Therefore, A-31, B-31, C-31, and D-31 were identified as the optimal ratios.
2. At 3% cement dosage, the distribution characteristics of the UCS curves of specimens at different ages are similar. The UCS value under the same replacement rate *R* increases with age, but the growth amplitude gradually decreases, indicating that the chemical reaction of gelatinous material gradually weakens, and the strength begins to stabilize. When *R* = 3%, the UCS values of the specimens at different ages reach their peak, and A-31 was determined to be the optimal ratio.
3. At the same cement dosage, the UCS of the specimen increases first and then decreases with the AOD slag replacement rate *R*. When *R* = 3%, the UCS of the specimen reaches its maximum of 4.4 MPa at 7 days, 9.3 MPa at 28 days, and 12.8 MPa at 60 days, indicating that the incorporation of AOD slag promotes the chemical reaction between cement and FA, forming a better skeleton structure, which plays a key role in improving the strength of the mixture. When *R* = 6% and *R* = 9%, the UCS of the specimen shows a downward trend, indicating that too much AOD slag is not conducive to the chemical reaction between cement and FA, and the strength decreases. When *R* = 12%, the AOD slag completely replaces the FA, and the UCS of the specimen reaches a minimum, indicating that the excitation effect of AOD slag is reduced.

**Figure 5:**
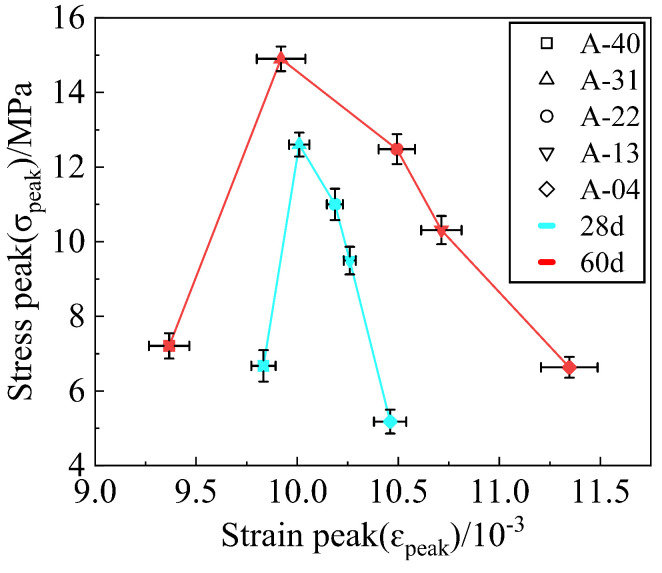
The graphical representation of stress peak and strain peak data for the mixture.

**Figure 6:**
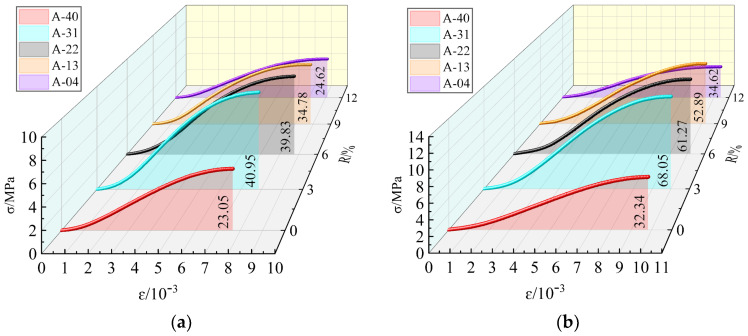
Stress strain curves of stainless steel AOD slag mixture. (**a**) Ages = 7 days and (**b**) ages = 28 days.

In order to eliminate the influence of differing stress strain results of the mixture as much as possible, each group of tests was designed to involve three samples, and the selected stress strain curve is the intermediate value of the three samples. Figure 5 shows the peak stress and peak strain data of the mixture specimens with the 3% cement dosage at 28 days and 60 days under different replacement rates, and the stress strain curve is shown in Figure 6. Incorporating stainless steel AOD slag into the cement FA-stabilized RA specimens enhances the UCS and ductility to varying degrees. The failure strain of the specimens increases with an increase in *R*, indicating that the expansive properties of stainless steel AOD slag play a vital role in improving the brittle behavior of the cement FA-stabilized RA specimens. Material toughness refers to a specimen’s ability to resist crack propagation, which can be assessed using the area under the stress strain curve of the specimen [43]. The results demonstrated that specimens A-31, A-22, A-13, and A-04 exhibited higher toughness than specimen A-40. The toughness of the mixture initially increased and then decreased with the increasing AOD slag replacement rate *R*, highlighting the advantage of stainless steel AOD slag as a road base material for withstanding cyclic vehicle loads.

### 4.3. Indirect Tensile Strength (ITS)

**Figure 7:**
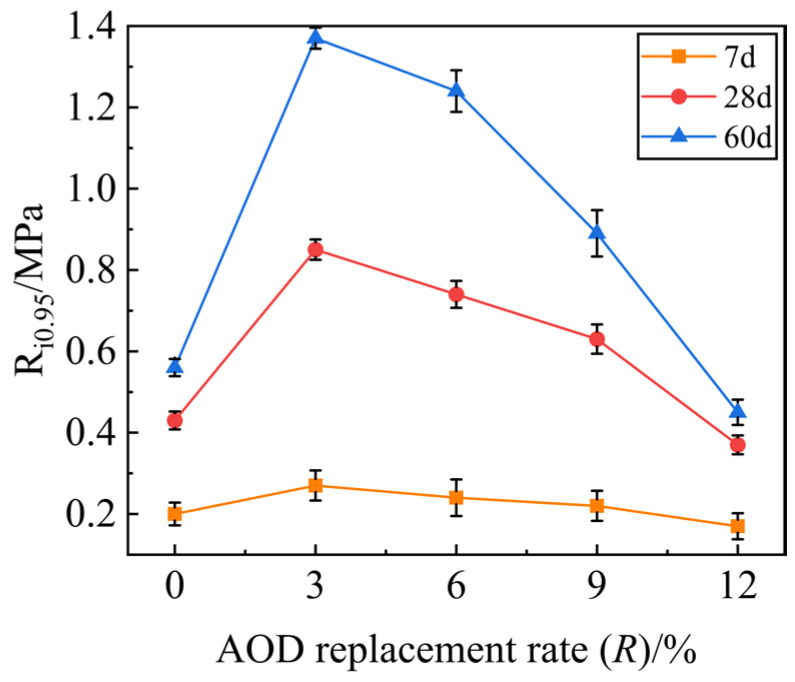
Indirect tensile strength of stainless steel AOD slag mixture at different ages.

Figure 7 shows the variation curve of the ITS representative value R_i0.95_ with an AOD slag replacement rate *R* at different ages of mixture specimens with the 3% cement dosage. The distribution characteristics of the ITS variation curves of mixture specimens at different ages are similar. The ITS under the same replacement rate *R* increases with the increase in age, and the growth amplitude decreases gradually, which is consistent with the distribution law of the UCS of the specimen. The ITS of the specimens show an increasing and then decreasing trend with the increase in stainless steel AOD slag replacement rate *R*, indicating that the stainless steel AOD slag directly affects the UCS and ITS of the mixture specimens. When *R* = 3%, the specimens reach their peak ITS, with maximum strengths of 0.27 MPa at 7 days, 0.85 MPa at 28 days, and a significant value of 1.37 MPa at 60 days. This suggests that the inclusion of the AOD slag enhances the ability of the mixture to withstand lateral deformation under stress.

**Figure 8:**
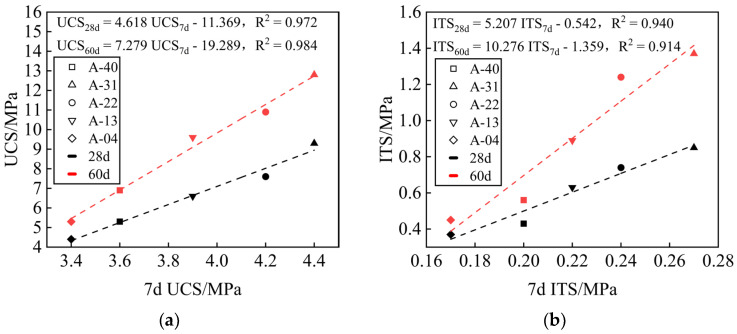
Strength growth model. (**a**) Relationship between 7-day, 28-day, and 60-day UCS results of the mixes at different replacement rates; (**b**) relationship between 7-day, 28-day, and 60-day ITS results of the mixes at different replacement rates.

Figure 8 shows the linear regression analysis of UCS and ITS at the 7-day, 28-day, and 60-day ages of the mixture. The UCS and ITS development trends with time under different replacement rates *R* are the same, with a good correlation. The strength growth is mainly determined by the hydration products. The UCS and ITS of the mixture at 28 days and 60 days can be estimated by the 7-day UCS and ITS results.

**Figure 9:**
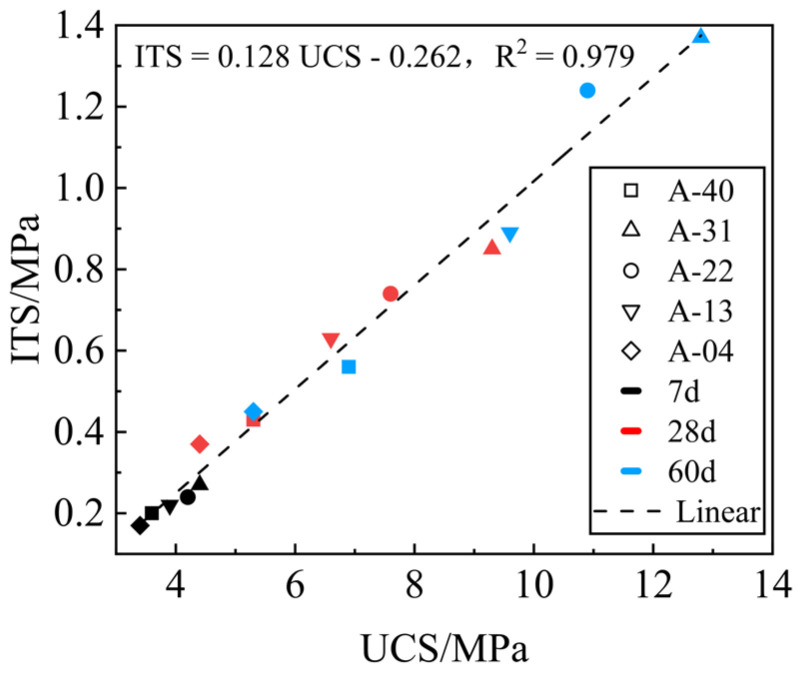
Relationship between the UCS and ITS of the mixes at different replacement rates.

The ITS test is usually used to evaluate material tensile or fracture resistance [44], but this test is more complicated than the UCS test. Therefore, establishing a correlation between the UCS and ITS and estimating the growth trend of ITS based on UCS using linear regression analysis can reduce costs and save time in road engineering. The fitting curve is shown in Figure 9.

### 4.4. Compressive Resilience Modulus (CRM)

**Figure 10:**
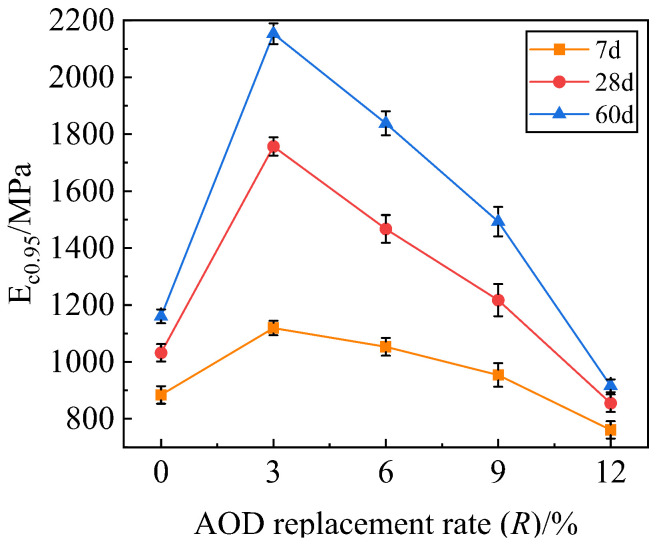
Compression resilience modulus of stainless steel AOD slag mixture at different ages.

Figure 10 shows the CRM representative value E_c0.95_ curve with the AOD slag replacement rate *R* at different ages of mixture specimens with the 3% cement dosage. The distribution characteristics of the CRM change curve of mixture specimens at different ages are similar. The CRM under the same replacement rate increases with age, and the growth amplitude decreases gradually. With an increase in the replacement rate *R* of stainless steel AOD slag, the CRM shows a trend of increasing first and then decreasing. When *R* = 3%, the CRM of the A-13 specimen reaches its peak. The maximum modulus at 7 days is 1119 MPa, the maximum modulus at 28 days is 1757 MPa, and the maximum modulus at 60 days is 2153 MPa. It shows that the hydration reaction is still occurring, and a large number of hydration products are produced to increase the bonding force between the mixtures and reduce the deformation caused by the force.

**Figure 11:**
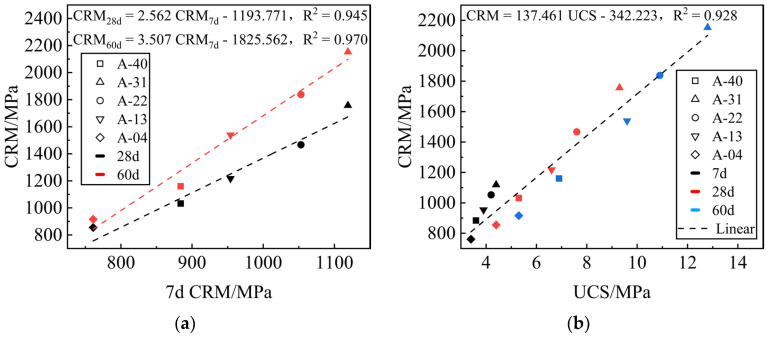
Modulus growth model. (**a**) Relationship between the 7-day, 28-day, and 60-day CRM results of the mixtures at different replacement rates; (**b**) relationship between UCS and CRM of the mixtures at different replacement rates.

Figure 11a shows the CRM linear regression analysis of the mixture at 7 days, 28 days, and 60 days. The CRM development trend with age is the same under different replacement rates *R*, and there is a good correlation. The 28-day and 60-day CRM can be estimated by the 7-day CRM. The CRM test is conducted during the material’s elastic deformation stage to measure the material’s mechanical properties in this stage, while the UCS test is carried out during the material’s plastic deformation stage to determine the material’s failure-bearing capacity [45]. The mechanics and deformation processes of the CRM test are included in the UCS test. Therefore, linear regression analysis was conducted between the UCS and CRM for the mixture specimens to indirectly monitor the compliance of construction and design, providing a theoretical basis for ensuring the quality of the project. The fitting curve is illustrated in Figure 11b.

### 4.5. Expansion Rate (ER)

**Figure 12:**
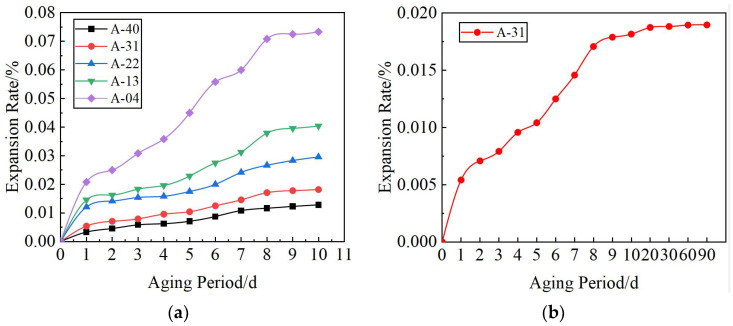
Water immersion expansion rate. (**a**) Expansion ratio of stainless steel AOD slag mixture; (**b**) optimum mix ratio of stainless steel AOD slag mixture sample expansion ratio.

Figure 12a shows the expansion rate curve of a 3% cement dosage mixture specimen under different replacement rates *R*. With the increase in high-temperature water bath time, the expansion rate also increases; eight days later, the rate tends to be gentle, and at 10 days, the expansion rates are 0.013%, 0.018%, 0.03%, 0.04%, and 0.073%, which meet the requirements for standard GB/T 25824-2010 [46] for steel slag used in road bases (expansion rate of less than 2.0%). The expansion rate of the specimen increases with the incorporation of AOD slag. When *R* = 3%, the expansion rate increases by 0.005%. When *R* = 12%, the expansion rate increases by 0.06%. The optimal ratio A-31 specimen was selected for continuous observation for 90 days. Under the continuous excitation of a high-temperature water bath, the expansion rate was 0.019% at 30 days, and there was no change at 90 days, which demonstrates good long-term stability. The growth curve is shown in Figure 12b.

### 4.6. X-ray Diffraction (XRD) Phase Analysis

**Figure 13:**
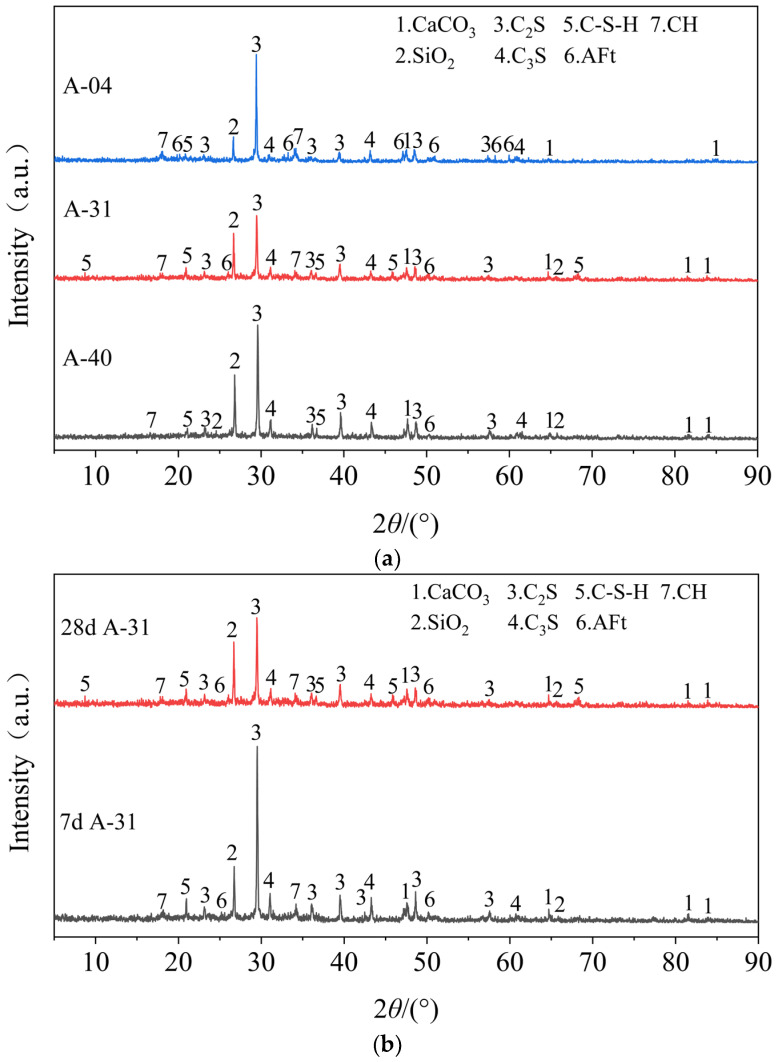
XRD phase analysis. (**a**) XRD patterns of hydration products of specimens A-40, A-31, and A-04 after 28 days of maintenance; (**b**) XRD patterns of hydration products of A-31 specimen after 7 and 28 days of maintenance.

The effect of AOD slag on the growth process of the hydration products of the mixture was explored. Figure 13a shows the XRD phase analysis spectrum of the mixture specimens (A-40, A-31, and A-04) aged 28 days at the 3% cement dosage. The X-ray diffraction patterns of all three specimens show the presence of AOD slag, as well as the original mineral components of cement such as dicalcium silicate (C_2_S) and tricalcium silicate (C_3_S), and the generated hydration products, including calcium hydroxide (CH), calcium silicate hydrate (C-S-H) gel, and ettringite (AFt). By comparing *R* = 3% and *R* = 0%, it was found that the characteristic AFt peak increased, and the characteristic C-S-H peak increased in both intensity and quantity. When *R* = 12%, the AOD completely replaced the FA, resulting in increased intensity and quantity of the AFt characteristic peak, a decrease in the characteristic peak of the C-S-H gel, and a decrease in quantity. This indicates that adding AOD slag promotes the rate of hydration and pozzolanic reactions. The abundant C-S-H gel filling in the voids of the mixture increases in density, which is the main reason for strength development. However, excessive AOD slag hampers the secondary hydration reaction. The accumulated CH in the later stages is not consumed, resulting in a decrease in hydration products and strength. The phase analysis elucidates the development patterns in the strength and modulus of the mixture.

Figure 13b shows the XRD patterns of the optimal ratio A-31 mixture specimens cured for 7 days and 28 days. By comparing the XRD patterns of the 28-day cured specimens with the 7-day cured specimens, a significant decrease in the characteristic C_3_S peak can be observed. In contrast, the decrease in the characteristic C_2_S peak is relatively small. This is because the hydration rate of C_3_S is faster, while the hydration rate of C_2_S is slower in the early stages, and its peak value also decreases with age. This indicates that most of the C_2_S and C_3_S in A-31 have reacted, resulting in increased intensity of the C-S-H gel diffraction peak. Furthermore, the characteristic peak of SiO_2_ in A-31 also shows a slight decrease. This is due to the presence of a certain amount of free calcium oxide (f-CaO) as an active component in the AOD. The f-CaO undergoes dissolution reactions with SiO_2_ in cement and FA in the presence of free water, reducing the formation of CH through hydration. This reduction in CH formation reduces the expansion of the samples. It reveals a pattern of initial expansion growth followed by stable expansion in the later stages.

### 4.7. Scanning Electron Microscopy (SEM) Morphology Analysis

**Figure 14:**
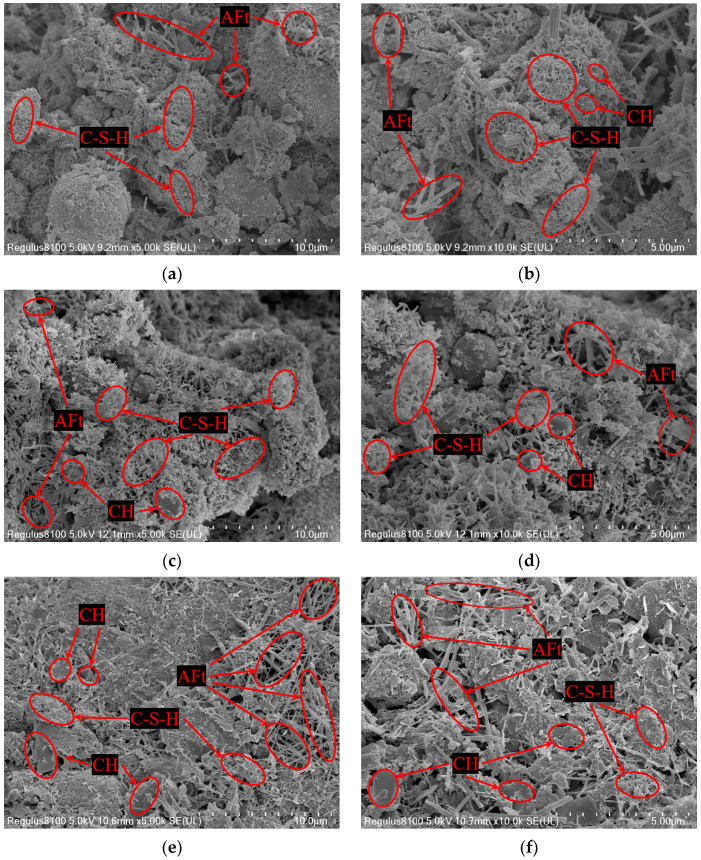
SEM patterns of hydration products of specimens A-40, A-31 and A-04 after 28 days of aging. (**a**) A-40, *R* = 0%, 5000 magnification; (**b**) A-40, *R* = 0%, 10,000 magnification; (**c**) A-31, *R* = 3%, 5000 magnification; (**d**) A-31, *R* = 3%, 10,000 magnification; (**e**) A-04, *R* = 12%, 5000 magnification; (**f**) A-04, *R* = 12%, 10,000 magnification.

According to the results of the XRD phase analysis, the internal structure change rule of the mixture with the addition of AOD slag was further explored, and the microstructure change of the AOD slag mixture sample was analyzed using scanning electron microscopy (SEM). Figure 14 shows the morphology characteristics of 5000 and 10,000 magnification scanning electron microscope images of the 28-day mixture specimens (A-40, A-31, and A-04). The overall structure of the mixture is mainly composed of the skeleton formed by RA extrusion and inlay stacking. The chemical reaction products between cement, FA, and AOD slag provide the cohesive strength for the base material. When *R* = 0%, the hydration reaction of cement produces CH crystals with a smooth surface and various shapes, reticular clusters of flocculated C-S-H gel, and a small amount of needle-like AFt [47]. In an alkaline environment, FA reacts with volcanic ash to form C-S-H gel, promoting the cement’s secondary hydration. The filling of pores by cementitious materials enhances the friction between aggregates. When *R* = 3%, under the excitation of AOD slag, the hydration reaction and pozzolanic reaction are more sufficient, and the C-S-H gels and AFt produced increase significantly compared with *R* = 0%. A large amount of gel material is filled between the aggregate pore gap and AFt, which effectively reduces the sample’s porosity and increases the mixture’s compactness. Cementation between particles makes the mixture’s structure more stable and greatly enhances its strength. When *R* = 12%, the AOD slag completely replaces the FA and cement, and undergoes hydration reactions; no volcanic ash reaction occurs, enriching a large number of CH crystals and AFt. Cement and AOD stop secondary hydration, the C-S-H gel is greatly reduced compared with *R* = 3%, and there is no colloidal hydration product around the dense AFt. The pores of the mixture are not filled, the compactness is reduced, and the failure strength is reduced. The development law and mechanism of strength and modulus initially increase and then decrease with the replacement rate *R*.

**Figure 15:**
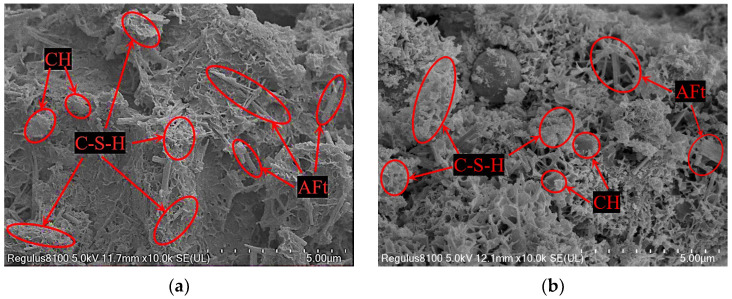
SEM patterns of hydration products of A-31 specimen after 7 days and 28 days of aging. (**a**) 7-day, 10,000 magnification; (**b**) 28-day, 10,000 magnification.

Figure 15 shows the SEM images of hydration products of the optimal ratio A-31 sample cured for 7 and 28 days. With increasing age, the morphology of the mixture sample changes from a loose structure to a dense structure. After curing for 7 days, the structure of the mixture is relatively loose, and there are many pores and cracks. The strength mainly comes from the large bite friction and sliding friction provided by the mutual extrusion of RA. Since a series of chemical reactions between cement, AOD slag, and FA are not completely carried out, only a small amount of C-S-H gel is formed and attaches to the surface of the FA and RA, and the strength of the mixture is low. When curing for 28 days, the hydration reaction between cement and AOD slag gradually becomes intense. In an alkaline environment, it will continue to react with FA to form a large amount of C-S-H gel, which fills between the interface transition zone of AFt and aggregate particles, completely wrapping the mixture, and supporting the skeletal structure of the mixture, which greatly improves its strength. Thus, the law and mechanism with which the strength and modulus of the mixture increases with age are revealed.

## 5. Conclusions

This study presents the research results of experiments with stainless steel AOD slag as a base material mixture, and the associated strength formation mechanism was analyzed in detail. Experimental studies of the UCS, ITS, CRM, and expansion rates of the mixtures at different substitution rates and different ages were carried out, and micro-mechanism analysis was conducted using SEM and XRD to explore the intrinsic laws of strength, modulus, and expansion rate of AOD slag mixtures in depth. The following important conclusions were obtained:

1. The compaction tests found that the OWC of the 20 sets of stainless steel AOD slag mixture ratios remained between 7% and 12%, while the MDD ranged from 1.96 to 2.09. The OWC increased with increasing *R*, while the MDD decreased with increasing *R*.
2. Based on the 7d UCS, a cement dosage of 3% was determined to meet the practical engineering requirements. The strength and modulus of the AOD slag mixture initially increased and then stabilized with age. The strength and modulus initially increased and then decreased with increasing *R*. According to the experimental results, it was determined that A-31 (*R* = 3%) was the optimal ratio. By establishing the regression relationship between the UCS, ITS, and CRM, it was found to be highly reliable, and it can be used to predict the strength and modulus of the road base.
3. The incorporation of AOD slag increases the expansion rate of the mixture. The 10d constant temperature water bath heating expansion rate of the mixture specimens under different *R* meets the GB/T 25824–2010 standard [44]. The expansion rate of steel slag for road base is less than 2.0%, its minimum value is 0.013%, its maximum value is only 0.073%, and after eight days, it tends to level off. Under the excitation of a high-temperature water bath for 90 days, the expansion rate of the optimal ratio A-31 sample is only 0.018%, and under later observation, it tends to stabilize.
4. According to microstructural analysis, after the mixture is statically pressure molded, RA becomes embedded with itself to form the basic skeleton of the material. The incorporation of AOD slag greatly promotes the hydration reaction and pozzolanic reaction. A large amount of C-S-H filled and cemented the interfacial transition zone between the RA, making the mixture structure denser, and increasing the interlocking friction and sliding friction in the RA. The ultimate strength of the mixture is enhanced, and the macroscopic laws of strength, modulus, and expansion rate are verified.
5. Through the above four conclusions, it can be concluded that the road performance of an AOD slag and recycled aggregate mixture is good, and this study provides new ideas and methods for its application in a road base. Currently, due to transportation costs, the road test section has not been completed. Next, numerical simulation methods will be further combined with road test section experiments for comparative analysis.

## Figures and Tables

**Table 1:** Performance indexes of cement.

SiO_3_/%	MgO/%	Loss/%	Initial Setting Time/min	Final Setting Time/min	Stability	3d Flexural Strength/MPa	28d Flexural Strength /MPa	3d Compressive Strength /MPa	28d Compressive Strength /MPa
2.3	3.2	3.9	188	235	Qualified	5.8	8.4	24.5	49

**Table 2:** Performance indexes of fly ash.

SiO_2_/%	Al_2_O_3_/%	Fe_2_O_3_/%	Loss/%	0.3 mm Sieve Passing/%	0.075 mm Sieve Passing/%	Water Content/%
45.1	24.2	0.85	2.8	100	89.6	0.85

**Table 3:** Chemical composition of stainless steel AOD slag.

CaO /%	SiO_2_ /%	Fe_2_O_3_ /%	MgO /%	TiO_2_ /%	Cr_2_O_3_ /%	MnO /%	K_2_O /%	SO_3_ /%
47.38	26.20	13.80	5.29	3.39	2.31	1.22	0.18	0.11

**Table 4:** Road performance indexes of recycled coarse aggregates.

Apparent Density/(g/cm^3^)	Crushing ^1^/%	Los Angeles Abrasion/%	Needle Flake Content ^2^/%	Water Content/%	Water Absorption/%	Impurity Content ^3^/%	Clay Content/%
2.685	23.3	26.1	16	1.15	2.6	0.4	0.8

^1^ Crushing: This refers to the performance index of the crushing strength and the crushing capacity of the crushed stone under a gradually increasing load. ^2^ Needle flake content: This refers to the percentage of slender needle-like particles and flat flake particles in the coarse aggregate to the total mass of the sample. ^3^ Impurity content: This refers to the percentage of wood chips, plastic, and other impurities present in of the recycled aggregate.

**Table 5:** Road performance indexes of recycled fine aggregate.

Fineness Modulus	Crushing/%	Water Absorption/%	Water Content/%	Clay Content/%
2.4	25.7	2.9	1.85	1.7

**Table 6:** Grading median of recycled aggregate.

Sieve Aperture Size/(mm)	Mass Percentage Passing through Different Sieve Apertures							
	31.5	19	9.5	4.75	2.36	1.18	0.6	0.075
gradation upper limit	100	98	70	50	38	27	20	7
gradation lower limit	100	81	52	30	18	10	8	0
gradation median	100	89.5	61	40	28	18.5	14	3.5

**Table 7:** Cement fly ash stainless steel slag stabilized recycled aggregate mix ratio.

Specimen Number	Composition Ratio of the Mixture			Specimen Number	Composition Ratio of the Mixture		
A-40, *R* = 0%	3% Cement	12% FA + 85% RA	85% RA	C-40, *R* = 0%	5% Cement	20% FA + 75% RA	75% RA
A-31, *R* = 3%		9% FA + 3% AOD		C-31, *R* = 5%		15% FA + 5% AOD	
A-22, *R* = 6%		6% FA + 6% AOD		C-22, *R* = 10%		10% FA + 10% AOD	
A-13, *R* = 9%		3% FA + 9% AOD		C-13, *R* = 15%		5% FA + 15% AOD	
A-04, *R* = 12%		12% AOD		C-04, *R* = 20%		20% AOD	
B-40, *R* = 0%	4% Cement	16% FA	80% RA	D-40, *R* = 0%	6% Cement	24% FA	70% RA
B-31, *R* = 4%		12% FA + 4% AOD		D-31, *R* = 6%		18% FA + 6% AOD	
B-22, *R* = 8%		8% FA + 8% AOD		D-22, *R* = 14%		10% FA + 14% AOD	
B-13, *R* = 12%		4% FA + 12% AOD		D-13, *R* = 18%		6% FA + 18% AOD	
B-04, *R* = 16%		16% AOD + 80% RA		D-04, *R* = 24%		24% AOD	

**Table 8:** Cement fly ash stainless steel slag stabilized recycled aggregate mix ratios.

Specimen Number	OWC ^1^/%	MDD ^2^/(g/cm^3^)	Specimen Number	OWC/%	MDD/(g/cm^3^)
A-40, *R* = 0%	7.7	2.082	C-40, *R* = 0%	8.4	2.059
A-31, *R* = 3%	8.7	2.066	C-31, *R* = 5%	9.3	2.046
A-22, *R* = 6%	9.6	2.053	C-22, *R* = 10%	10.3	2.031
A-13, *R* = 9%	10.2	2.041	C-13, *R* = 15%	10.8	2.017
A-04, *R* = 12%	10.9	2.030	C-04, *R* = 20%	11.3	1.999
B-40, *R* = 0%	8.1	2.069	D-40, *R* = 0%	8.7	2.041
B-31, *R* = 4%	9.0	2.058	D-31, *R* = 6%	9.6	2.021
B-22, *R* = 8%	10.0	2.043	D-22, *R* = 14%	10.5	1.998
B-13, *R* = 12%	10.5	2.030	D-13, *R* = 18%	10.9	1.976
B-04, *R* = 16%	11.1	2.016	D-04, *R* = 24%	11.5	1.960

^1^ OWC: optimum water content. ^2^ MDD: maximum dry density.

## Data Availability

The data presented in this study are available upon request from the corresponding author.
